# Determinants of Intravascular Resistance in Indian Diabetic Nephropathy Patients: A Hospital-Based Study

**DOI:** 10.1155/2011/656030

**Published:** 2011-06-12

**Authors:** Anubhav Thukral, Manish Mishra, Vaibhava Srivastava, Hemant Kumar, Amit Nandan Dhar Dwivedi, Ram Chandra Shukla, Kamlakar Tripathi

**Affiliations:** ^1^Department of Medicine, Institute of Medical Sciences, Banaras Hindu University, Varanasi 221005, India; ^2^Department of Radiodiagnosis, Institute of Medical Sciences, Banaras Hindu University, Varanasi 221005, India

## Abstract

*Aims and Objectives*. Metabolic dysregulation has failed to explain clinical variability of patients with diabetic nephropathy and hence a renewed interest emerged in haemodynamic factors as determinant of progression and development of diabetic nephropathy. We therefore studied for various factors which can correlate with raised renal vascular resistance in diabetic nephropathy. *Material and Methods*. Renal vascular resistance was measured in patients with established and incipient diabetic nephropathy and compared with controls using noninvasive color Doppler examinations of intrarenal vasculature. *Results*. Renal vascular resistance correlated with age, duration of disease, GFR, serum creatinine, and stage of retinopathy. Renal vascular resistance was significantly reduced in patients on treatment with RAAS inhibitors and insulin, than those on OHA and antihypertensives other than RAAS inhibitors. *Conclusion*. The study implies that renal vascular resistance may help identify diabetics at high risk of developing nephropathy, and these set of patients could be candidates for RAAS inhibition and early insulin therapy even in patients without albuminuria.

## 1. Introduction

Diabetes and its complications pose an immense amount of social and economic burden on the health infrastructure and resources throughout the globe. Diabetic nephropathy is the single most common cause of end-stage renal disease (ESRD) throughout the globe accounting for a whooping 25–45% of all patients enrolled in ESRD programmes [1]. Diabetic nephropathy is also a leading cause of chronic kidney disease (CKD) in India accounting for about 30% of all CKD patients [2]. Recent estimates suggest that soon India, China, and United States are and will remain the countries with largest number of diabetics [3].

In spite of several decades of research since 1940's when several studies linked diabetes to renal disease [4, 5], there are still large gaps in the knowledge and understanding of pathophysiology of diabetic nephropathy.

One of the intriguing controversies has been that which patients of diabetes (type 1/type 2) are predisposed and are likely to progress to diabetic nephropathy. Several studies have demonstrated that only about 40% of patients of Type 1 and Type 2 diabetes have renal involvement. These set of patients that progress to frank diabetic nephropathy have been labeled as “progressors” and the other set of patients that in spite of similar control of blood sugar and long-term poor glycemic control do not progress have been labeled as “nonprogressors” [6].

There has been a paradigm shift in the understanding of factors held responsible for this discrepancy in the natural history of these two sets of patients with the balance tilting towards hemodynamic factors rather than towards metabolic factors [7]. The other, controversy in diabetic nephropathy surrounds the concept of microalbuminuria. Albuminuria has been linked to diabetic renal disease as early as 1836 by Bright [8]. Several studies have established microalbuminuria as a hallmark of diabetic nephropathy and microalbuminuria has been used as a predictive marker for progressive decline in renal function [9–11], however few researchers have challenged its significance especially after studies showed that many patients of diabetic nephropathy with advanced renal disease never develop albuminuria [12–14] and also 58–68% patients with microalbuminuria regress over a period of time to normoalbuminuria [15, 16].

Another area of active research has been finding suitable markers for predicting which patients will progress. Many studies addressed biochemical markers but none is specific or sensitive enough for clinical use [17–19].

Of late researchers have tried to answer all these controversies by a single key and that is hemodynamic alterations [20] which include concepts of intraglomerular hypertension, raised renal vascular resistance and so called ischemic nephropathy, which have been held responsible for discrepancy in behavior of various groups; recently an article by Nosadini et al. established renal vascular resistance as a predictive marker for progressive disease [6].

Prompted with above considerations, we designed our study to evaluate renal vascular resistance with noninvasive Doppler in 57 diabetic patients attending a tertiary care centre in India.

## 2. Material and Methods

This study was carried out in the Department of Medicine and Radiology, Sir Sunderlal Hospital, Institute of Medical sciences (IMS), Banaras Hindu University (BHU) from May 2008 to June 2010. The study protocol was approved by the research and ethical committee of the institute and written informed consent was taken from all patients.

A total 31 patients of diabetic nephropathy with stable disease, and three groups of control subjects (19 healthy subjects, 14 diabetics without microalbuminuria, and 12 diabetics with microalbuminuria) were included in the study. Diabetes was diagnosed as per WHO guidelines [21]. All of our patients were of type 2 diabetes. Diabetic nephropathy was labeled clinically satisfying the following criteria: (a) evidence of retinopathy on fundus examination, and (b) persistent albuminuria > 300 mg/24 hrs or persistent proteinuria > 500 mg/dl on urine analysis on two separate occasions 3 months apart.

Microalbuminuria defined as 24 hr urinary albumin 30–300 *μ*g/dl. 24 hr urinary collection was done from 8 AM to 8 AM next day with morning urine of day of start of collection discarded and morning urine of next morning collected. Adequacy of collection was confirmed by 24 hr urinary creatinine estimation wherever necessary. Urine routine microscopy and urine cultures were done before estimation of 24 hr urinary protein to rule out urinary tract infection. Patients with diabetic foot, valvular heart disease, ischemic heart disease, glomerulonephritis, lupus nephritis, renal calculi, hydronephrosis, renal masses, cerebrovascular, cardiovascular, and peripheral vascular disease were excluded.

### 2.1. Doppler Study

1. Pulsed Doppler study of interlobar and arcuate arteries was performed on bilateral kidneys.

**Resistive Index (RI)**

Peak systolic frequency shift—minimum diastolic frequency shift/Peak systolic frequency shift during whole cardiac cycle.

**Pulsatility Index (PI)**

Peak systolic frequency shift—minimum diastolic frequency shift/Mean frequency during the whole cardiac cycle.

We used a Toshiba NEMIO-30 Doppler machine and recorded findings with the help of a curvilinear probe of 3.75 Mhz. The sonologist was unaware of the group to which the patient belonged. RI and PI readings 2 each per vessel (interlobar arteries) for both kidneys were recorded and average of all 4 readings was taken for study purposes. 

B mode ultrasound was also done to look for renal size length, breath, and thickness. Patients with anatomical renal artery stenosis of the renal artery (main) were excluded from the study. Wall filter was set to a minimum of 50 Hz and with a Doppler sample volume set at 2–4 mm, minimum pulse repetition frequencies that did not produce aliasing were used with readings noted from existing software in the machine and all readings done at minimum possible angle between ultrasound beam and the vessel. Patients were rested for 5 min before taking any Doppler reading and all readings made while subject was lying in supine position with the breath held in deep end inspiration. Subjects were required to hold their breath while readings were being taken to obtain adequate spectral waveforms. Readings of RI and PI were made after a minimum of 4 identical consecutive spectral waveforms were achieved. Reproducibility of Doppler readings was assessed by intraobserver and interobserver variance which was 3.9% and 5.0%, respectively.

Many subjects could not cooperate in the study and could not hold breaths adequately and were removed from the study especially patients with morbid obesity, respiratory problems, and so forth. This may be the reason for a nonintentional lower mean BMI values in our study population.

Blood pressure was recorded after a 5 min rest just before the Doppler examination in right arm supine position and a second reading was made 5 min after the Doppler examination was completed. Average values of both readings were recorded. Mean blood pressure was calculated for comparison purposes. A standard health questionnaire was completed which included type, duration of diabetes, treatment history, and other significant illness like hypertension and coronary artery disease were also enquired into.

Patients with proteinuria had to have proteinuria on two occasions three months apart; they were put on adequate and necessary treatment in the first visit and most of them continued treatment till the next visit, therefore labeling a patient as “on insulin” or “on OHA” or “on ACE/ARB” meant they were on that treatment for at least 3 months. 

Other patient variables like age, 24 hour urinary protein/albumin, HbA1c% values, fundus examination, lipid profile, and anthropometric measurements were also recorded. Serum glucose level (FBS, PPBS) was estimated by glucose oxidase/glucose peroxidase method and Serum lipid parameters (total cholesterol, HDL-C, LDL-C, very low density lipoprotein, and triglyceride) were quantified by commercially available Kits (Span diagnostics, India) using manufacturer's protocol using Autoanalyzer (Flexor XL machine, Vitro sciences). HbA1c% was measured in venous sample after an overnight fast by D10 Bio-rad system automated machine using HPL-C technique of electrophoresis. All the biochemical tests were performed in hospital's central biochemical laboratory.

Fundus examination was done by an ophthalmologist who did not know the group under which the patient felt. Staging of the disease was done according to International Clinical Diabetic Retinopathy Disease Severity Scale. We used the GAULT COCKROFT FORMULA to calculate GFR.

## 3. Statistical Analysis

All the data were reported as mean ± standard deviation if they were normally distributed and as median ± Inter Quartile Range (IQR) if they were not normally distributed. One way ANOVA was applied when comparing parametric variables (mean) between four groups. Kruskal-wallis one way Anova was used to compare median values between groups if data was not normally distributed. Pearson's correlation was used to see corelation of RI and PI with various variables. Multivariate linear and stepwise forward progression analysis was done using RI and PI as dependent variables and other variables as independent variables. Subgroup analysis was done using unpaired student's “*t*”-test between two groups. Chi square test was used for rates and proportions, however if >20% values were under 5, Fisher exact test was used. Mann Whitney test was used for nonparametric variables between two groups. *P*-value <.05 was ascribed as significant difference between groups. Data were analyzed by the sigma stat software version 3.5.

## 4. Results

The baseline characteristics showed that a significant difference between the groups existed in serum cholesterol, GFR, and treatment of hypertension. Patients with diabetic nephropathy and diabetics with microalbuminuria had higher levels of serum cholesterol than patients without microalbuminuria. GFR showed progressive decline from diabetes without albuminuria to diabetic nephropathy. Patients with diabetes without microalbuminuria although had mean values lower than control population but there were values above values in normal population signifying presence of hyperfiltration early in diabetes. Patients in diabetic nephropathy group had 9 patients receiving ACE/ARB, others were receiving other antihypertensive as either these patients had contraindications to use of this drug or could not tolerate the drug. Higher percentages of patients in diabetes with microalbuminuria group were on ACE inhibitors. Patients in this group who were not on ACE inhibitors were again those in whom it was contraindicated or not tolerated or patients who did not comply with the advised treatment. Duration of disease was not significantly different in the various groups signifying that in spite of long duration of diabetes many patients did not develop diabetic nephropathy and still remained in the group of diabetes without microalbuminuria, the median duration of disease in this group was 2.5 ± 19.25 yrs, which was nonsignificantly lower than other groups (Table 1).

The mean resistive index (RI) values of interlobar arteries showed a statistically significant difference (*P* < .001) between each group except between group B and group C that is no significant difference was seen between subjects with microalbuminuria and subjects without microalbuminuria. This would imply that RI values start increasing in diabetic subjects even before appearance of microalbuminuria.

The mean values of pulsatility index (PI) of interlobar arteries were also statistically significantly raised as compared to nondiabetics (*P* < .001) but intragroup differences were significant only between subjects with diabetic nephropathy and diabetics without microalbuminuria (Table 2).

RI and PI on univariate analysis correlated significantly with age, serum creatinine, GFR, stage of diabetic retinopathy, and treatment group for diabetes; patients on insulin having lower values of RI and PI, on multivariate linear analysis and multivariate stepwise forward progression using RI and PI as dependent variable RI and PI correlated with age, serum creatinine, and GFR (Table 3).

There was also significant co relation of stage of retinal disease with RI and PI values (*P* < .001) (Spearman co relation coefficient). On multiple comparison tests there was significant difference between RI values of stage 4 versus normal and Stage 2 versus normal; PI also correlated with stage of fundal disease with higher values in higher retinal stage (*P* < .001). On multiple comparison tests there was significant difference between PI values of stage 4 versus normal, Stage 3 versus normal, stage 2 versus normal, and stage 2 versus stage 1.

Subgroup analysis of diabetic subjects on basis of RI values showed that the groups differed significantly only in the treatment received for diabetes with patients with significant proportion of patients with RI < 0.8 on Insulin therapy (Table 4). 

On classifying diabetic patients according to treatment received patients on insulin therapy had significantly lower values of RI than patients on OHA's in spite of having similar blood sugar controls in form of almost same HbA1c% levels. However fasting blood sugar levels were significantly higher in patients on insulin. Therefore in spite of equal or even poor glycemic control patients on insulin had lower RI values. PI values were also nonsignificantly lower in patients on insulin (1.33 versus 1.74) (Table 5).

## 5. Discussion

Our study did not show any co relation of RI or PI values with BMI, Sex, FBS, HbA1c, serum cholesterol, serum triglyceride, duration of disease, or mean blood pressure. Barring a few studies our results are in accordance with many similar studies on this topic. These findings suggest that there are factors other than the level of metabolic control that contribute to diabetic nephropathy and raised renal vascular resistance in these patients. Haemodynamic factors like blood pressure control could explain these variations but studies have shown conflicting results. Ishimura et al. [22] showed no correlation between RI and PI values and mean blood pressure however Kim et al. [23] showed a significant co relation of RI and PI values with mean blood pressure.

Our study showed significant co relation of RI and PI values with serum creatinine and eGFR. GFR of subjects with microalbuminuria was significantly lower than subjects without proteinuria. This emphasizes the point that microalbuminuria is not a very good marker for early detection of diabetic nephropathy since fall in GFR has already set in once microalbuminuria develops, and hence the need to identify a more early marker. 

RI and PI values of all diabetics were 0.805 ± 0.187 and 1.63 ± 0.831, which was significantly higher than that of controls. Intragroup comparison showed significant differences between groups except between groups with and without microalbuminuria, that is, RI was raised even before microalbuminuria started. There were 2 patients with serum creatinine > 1.5, that is, with already set-in renal failure but still no microalbuminuria. No correlation of RI or PI was found with amount of proteinuria on univariate analysis. Kim et al. [23] cited a significant correlation of 24-hour protein value with RI Hamano et al. [24] did not show any correlation with amount of proteinuria as a continuous variable on univariate analysis. However both Hamano et al. [24] and Ljubiæ et al. [25] showed significant correlation of RI with proteinuria on multivariate stepwise regression analysis.

Another issue that has been addressed in various studies is that diabetic nephropathy has been demonstrated to have higher renal vascular resistance than other causes of CKD [26]. This led to the postulation that there is some specific pathophysiology to diabetes that causes this raised renal vascular resistance. Although diabetic nephropathy has been classically described as a microvascular complication, however there is another school of thought that thinks that diabetic nephropathy and raised renal resistance are a part of accelerated diffuse atherosclerotic process and widespread endothelial dysfunction that accompanies diabetes. This is based on several observations that renal vascular resistance is particularly higher in patients with lower limb vascular disorders in diabetes. Also pathological studies have shown arterial sclerosis in kidney biopsy of medium-sized arteries perpendicular to the kidney surface. Therefore several studies have tried to correlate RI and PI with markers of macrovascular disease such as Carotid intimo-medial thickness (IMT) and aortic stiffness parameters like brachial ankle pulse wave velocity (ba-PWV) and ankle brachial pressure index (ABI). 

In our study we excluded patients with macrovascular complications and still found significant higher RI values. We also demonstrated a significant correlation of stage of retinal disease with RI & PI values which itself is a microvascular complication. There was significant difference between RI & PI values of stage 4 versus normal, stage 2 versus normal, and stage 2 versus stage 1. PI also corelated with stage of fundal disease with higher values in higher retinal stage. This suggests that raised renal vascular resistance starts due to diabetic microvascular pathophysiology and at a later date may get accelerated due to accompanying macro vascular complication.

Therefore we from above findings conclude that RI and PI as an indices of renal vascular resistance are an early marker for recognition of diabetic nephropathy and also are predictive factorsof recognition of patients at risk for future development of diabetic nephropathy. However this exercise is useful only if we have measures to retard progression of these patients to diabetic nephropathy. One of the useful interventions is ACE/ARB, which in numerous studies have shown beneficial effect on prognosis of diabetic nephropathy. In our study diabetic patients on ACE/ARB had significantly lower PI values than patients not on ACE/ARB and nonsignificantly lower RI values; in spite of similar age, blood pressure, and glycemic control.

Another important finding of our study was that patients on insulin had significantly lower RI and PI values, in spite of similar age, blood sugar and blood pressure control, and proteinuria. Several studies indicate that insulin deficiency per se contributes to the neovascularization, increased surface area and afferent arteriolar dilation in diabetes. Several studies show that physiological concentrations of insulin cause afferent arteriolar constriction and efferent arteriolar dilation. These observations strongly support the notion that insulin deficiency directly contributes, at least in part, to the glomerular hypertension in diabetes. Some studies have addressed differential class effect of antidiabetic drugs on renal vascular resistance. Of note is a study by Pistrosch et al. [27], which concluded that rosiglitazone an insulin sensitizer had beneficial effect on renal vascular resistance as well as endothelial dysfunction.

However our study has certain limitations in the form of small sample size, a hospital-based sample, and cross-sectional nature of the study; hence large longitudinal population-based studies are required before any clinically meaningful outcomes can be derived from our observations.

## Figures and Tables

**Table 1:** Baseline characteristics of different groups.

Variable	Diabetic nephropathy (Group A) (*n* = 31)	Diabetics with microalbuminuria (Group B) (*n* = 14)	Diabetics without microalbuminuria (Group C) (*n* = 12)	Nondiabetic controls (*n* = 19)	*P*-value
Age (yrs) Median ± IQR	58 ± 14.25	60 ± 15	58.50 ± 15	50.00 ± 22.25	.059 (NS)
Sex Ratio (M/F)	20/11	7/7	10/2	13/6	.351 (NS)
Duration of diabetes (yrs) Median ± IQR	7.00 ± 11.25	3.5 ± 11.75	2.5 ± 19.25	—	.179 (NS)
BMI (Kg/m^2^) Mean ± SD	22.416 ± 4.218	22.451 ± 4.007	24.008 ± 4.756	21.872± 3.974	.581 (NS)
Treatment diabetes (OHA/Insulin)	21/10	9/5	9/2	—	.602 (NS)
Treatment hypertension (ACE or ARB/others)	9/22	8/6	0/12	1/18	<.001
Serum Creatinine Median ± IQR	1.9 ± 2.1	1.2 ± 1.2	1 ± 0.4	0.9 ± 0.1	<.001
GFR (ml/min) Mean ± SD	37.134 ± 23.5	48.383 ± 21.042	71.456 ± 32.666	78.455 ± 18.96	<.001
FBS (mg/dl) Median ± IQR	170 ± 130.5	180 ± 100	157 ± 74.5	78 ± 15	<.001*
Hb1Ac (%) Median ± IQR	8.5 ± 3.15	8.0 ± 2.2	7.15 ± 1.55	4.1 ± 0.3	<.001*
Cholesterol (mg/dl) Median ± IQR	106 ± 28.5	102.5 ± 75	73.5 ± 44	73 ± 9.75	<.001**
Triglyceride (mg/dl) Median ± IQR	120 ± 126	127 ± 153	102 ± 97.5	88 ± 20	.144
Mean blood pressure (mmHg) Mean ± SD	96.75 ± 12.1	97.95 ± 10.5	92.5 ± 10.195	97.36 ± 16.16	.691 (NS)
Fundus staging (early/late) Early = stage 1&2 Late = stage 3&4	19/12	8/2*	3/0*	—	.256 (NS)

*There was no significant difference between groups A, B, and C.

**There was no significant difference between A and B, but significant difference between A and C, and between B and C.

**Table 2:** Comparison of RI and PI between groups.

	Diabetic nephropathy	Diabetics with microalbuminuria	Diabetics without microalbuminuria	Nondiabetic controls	*P*-value
RI Median ± IQR	0.825 ± 0.178	0.742 ± 0.21	0.74 ± 0.169	0.64 ± 0.097	<.001

PI Median ± IQR	1.86 ± 0.82	1.388 ± 0.67	1.263 ± 0.35	1.075 ± 0.39	<.001

**Table 3:** Correlation of RI and PI with various variables.

Index	Parameter	Correlation coefficient	*P*-value
RI	Age	0.329	.003
PI	Age	0.383	.000
RI	BMI	−0.0235	.840
PI	BMI	0.0221	.849
RI	Duration	−0.149	.266
PI	Duration	0.0005	.997
RI	Diastolic blood pressure	−0.002	.985
PI	Diastolic blood pressure	−0.032	.78
RI	Systolic blood pressure	0.0265	.820
PI	Systolic blood pressure	−0.0188	.871
RI	Mean blood pressure	0.001	.988
PI	Mean blood pressure	−0.0385	.740
RI	Fasting Blood sugar	−0.145	.281
PI	Fasting Blood sugar	−0.178	.183
RI	Cholesterol	−0.162	.227
PI	Cholesterol	−0.0475	.725
RI	Triglyceride	−0.143	.289
PI	Triglyceride	−0.129	.339
RI	Serum creatinine	0.368	.001
PI	Serum creatinine	0.479	.000
RI	GFR	−0.456	.000
PI	GFR	−0.534	.000
RI	Fundus stage	0.425	.001
PI	Fundus stage	0.525	.000

**Table 4:** Comparison of patients with or without raised vascular resistance.

Characteristics	R.I ≥ 0.8 (*n* = 30)	RI < 0.8 (*n* = 27)	*P* value
Age Mean ± SD	59.467 ± 10.837	56.11 ± 8.243	.198

Sex ratio (M/F)	19/11	18/9	.988

BMI Median ± IQR	21.458 ± 3.895	22.862 ± 6.431	.179

Duration Median ± IQR	5 ± 9	10 ± 19	.381

Mean B.P. (Mean ± SD)	95.289 ± 12.508	97.11 ± 10.005	.549

FBS Median ± IQR	160 ± 88	170 ± 88.5	.406

Fundus staging (early/late) Early = stage 0, 1&2 Late = stage 3&4	20/10	22/4	.216

GFR Mean ± SD	42.741 ± 24.043	50.79 ± 29.474	.261

Treatment category (OHA/Insulin)	26/4	14/13	.010

Treatment hypertension (ON ACE or ARB/others)	8/22	11/16	.399

**Table 5:** Comparison of patients between treatment groups.

Characteristics	On insulin*(*n* = 17)	On OHA* (*n* = 40)	*P*-value
Age Mean ± SD	57 ± 7.508	58.25 ± 10.636	.662

Sex ratio (M/F)	10/7	27/13	.745

BMI Mean ± SD	23.652 ± 4.014	22.381 ± 4.35	.307

Duration	10 ± 19.188	5 ± 11	.88

Mean B.P Mean ± SD	96.039 ± 11.699	96.2 ± 11.391	.961

Hb1Ac Median ± IQR	8.3 ± 2.7	7.55 ± 2.6	.341

FBS Median ± IQR	200 ± 117	160.5 ± 76.5	.140

Fundus staging (early/late) Early = stage 0, 1&2 Late = stage 3&4	13/4	30/10	1

GFR Mean ± SD	51.889 ± 32.596	45.725 ± 25.164	.443

Treatment hypertension (ON ACE or ARB/others)	8/9	11/29	.260

RI Median ± IQR	0.703 ± 0.117	0.835 ± 0.182	.001

PI Median ± IQR	1.33 ± 0.490	1.742 ± 0.974	.094

Proteinuria (mg/day) Median ± IQR	600 ± 2862	540.5 ± 648	.291
